# AeroGRT: Geometry-Aware Global–Local Routing Transformer with Query-Reliability Graphs for UAV Object Recognition in Complex Scenes

**DOI:** 10.3390/s26216743

**Published:** 2026-10-24

**Authors:** Yihua Zang, Xingmin Ma, Zhi Liu, Mengxing Ma

**Affiliations:** 1 System Department 2, 15th Research Institute of China Electronics Technology Group Corporation, Beijing 100083, China; maxingmin1985@163.com; 2 School of Information, Beijing Institute of Technology, Beijing 100081, China; 7520170087@bit.edu.cn; 3 School of Electrical and Information Engineering, Yunnan Minzu University, Kunming 650031, China; mamengxing1980@163.com

**Keywords:** UAV image recognition, object detection, transformer, cross-attention, query-reliability graph

## Abstract

**Highlights:**

**What are the main findings?**

- By integrating global–local dual encoding, cross-axis boundary attention, and query-reliability graph attention, AeroGRT achieved 75.8% average overlap (AO), 83.9% SR0.5, and 73.1% SR0.75 on GOT-10k, improving AO by 12.4 percentage points over the ablation baseline.
- AeroGRT attained 87.4% AUC, 91.3% normalized precision, and 87.6% precision on TrackingNet, while improving mAP50 by 2.8 and 1.1 percentage points on VisDrone and TinyPerson, respectively, compared with the corresponding YOLOv8-based baselines.

**What are the implications of the main findings?**

- The synergistic integration of boundary-sensitive feature fusion and feature-consistency-guided attention provides a geometry-aware mechanism for preserving fine-grained target representations and mitigating unreliable foreground–background associations under occlusion, scale variation, and severe visual clutter.
- The proposed scale–altitude routing strategy adaptively redistributes detection confidence across feature-pyramid levels without altering bounding-box geometry, offering a unified architectural foundation for multiscale UAV object perception.

**Abstract:**

UAV visual perception is increasingly important for intelligent transportation monitoring, infrastructure inspection, emergency response, and low-altitude surveillance. However, reliable target localization remains challenging in complex scenes where targets are small, densely distributed, weakly textured, partially occluded, or visually similar to the background. Motivated by attention-based feature fusion and the routing attention design of AnisoNet, this paper proposes AeroGRT, a framework integrating global–local feature encoding and query-reliability graph attention for UAV visual perception in complex scenarios. The proposed framework combines a CNN-based local encoder, a Transformer-based global encoder, an adaptive global optimization module, a foreground–background fusion attention module, a cross-axis boundary attention mechanism, a query-reliability graph attention module, and a scale–altitude routing detection head. The architecture is designed to integrate local spatial detail with long-range semantic context, preserve target boundary information, and reduce unreliable query–key associations between foreground targets and background clutter. AeroGRT was evaluated on GOT-10k and TrackingNet for object tracking and on VisDrone and TinyPerson for small-object detection, using task-specific protocols and metrics. These evaluations assess performance under occlusion, deformation, low contrast, background clutter, and long-range observation. This study investigates the joint modeling of local detail, global context, boundary cues, and query reliability for accurate UAV target localization in challenging environments.

## 1. Introduction

UAV platforms provide flexible low-altitude sensing, wide spatial coverage, and rapid deployment for traffic surveillance, road infrastructure inspection, urban security, agricultural monitoring, and emergency rescue. Public drone benchmarks such as VisDrone, UAVDT, and UAV123 have made it possible to evaluate detection and tracking algorithms under realistic UAV viewpoints, dense traffic scenes, motion blur, scale variation, and severe background clutter [1,2,3]. Compared with fixed surveillance cameras or ground vehicles, UAV imagery introduces stronger scale instability, larger perspective changes, and more frequent small-target conditions. These characteristics make UAV image recognition a demanding research problem that connects object detection, single-object tracking, multi-object tracking, semantic perception, and edge computing deployment.

The development of deep object detectors has substantially improved image recognition accuracy. Region-based approaches introduced candidate-region reasoning and high-quality localization [4,5], while one-stage detectors represented by the YOLO family advanced real-time recognition for practical deployment [6,7,8,9]. More recently, DETR-style detectors and real-time detection Transformers have improved the modeling of long-range dependencies and set-based prediction [10]. Nevertheless, UAV scenes remain challenging because target size, target density, viewpoint, and background texture change simultaneously. A detector that performs well in ground-level images may fail when the same target occupies only a few pixels, is partially occluded by vegetation or vehicles, or is embedded in shadows and textured roads.

Transformer models offer powerful global modeling ability because multi-head self-attention can directly establish dependencies among distant visual tokens [11,12,13,14,15,16,17]. In visual tracking, TransT [18] replaces correlation-based template–search feature fusion with an attention-based network comprising an ego-context augment module based on self-attention and a cross-feature augment module based on cross-attention. These developments motivate the investigation of attention-driven feature interaction for UAV visual perception. In this study, we focus on preserving local image details, representing object boundaries, and suppressing unreliable foreground–background associations. These challenges motivate the combination of local feature extraction, global contextual modeling, and query–key consistency modeling in AeroGRT.

**Figure 1:**
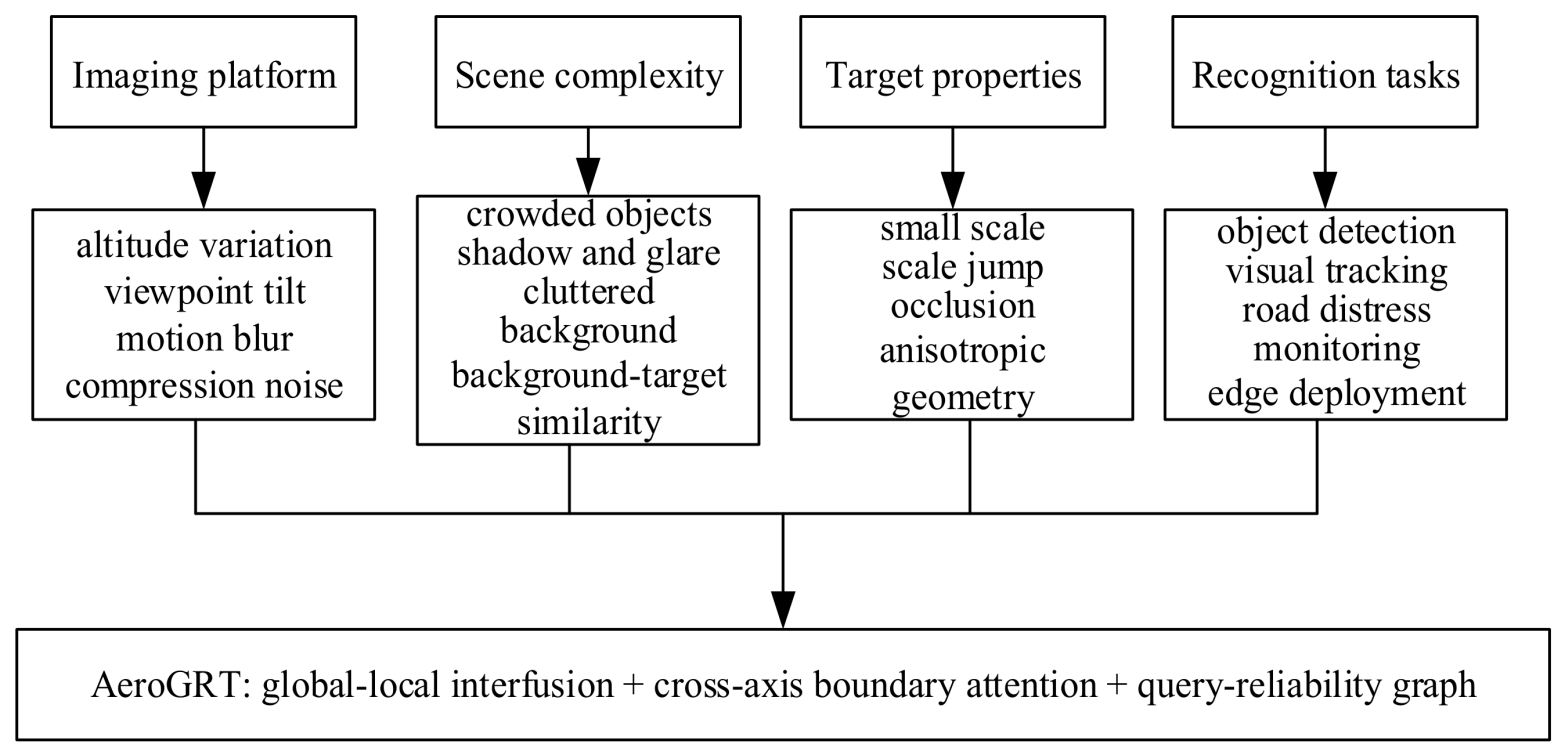
Complex UAV image recognition scenario taxonomy and the corresponding AeroGRT algorithm.

Crack-monitoring studies investigate low-contrast and geometrically irregular road defects in ground-based and UAV imagery [19,20,21,22,23,24,25,26]. AnisoNet [27] provides a related example of attention and convolutional designs for anisotropic crack detection. Such research motivates considering target geometry when developing feature representations for UAV images, in which small or elongated objects may have weak contours and limited visual detail. This study therefore investigates the integration of global–local feature encoding, boundary-sensitive attention, query-reliability modeling, and geometry-aware routing for UAV object localization. Figure 1 summarizes the challenging UAV scenarios considered in this study and their relationship to the AeroGRT framework.

The main contributions are summarized as follows: (1) We propose AeroGRT, an interfusion feature extraction and query-reliability graph Transformer for UAV image recognition in complex scenarios. (2) We design a dual-encoder global–local feature extraction network that preserves local detail while maintaining global semantic context. (3) We formulate a cross-axis boundary attention mechanism to fuse horizontal and vertical local information and enhance boundary-sensitive features. (4) We construct a query-reliability graph attention module that uses feature-space consistency to weight query–key associations.

The remainder of this paper is organized as follows: Section 2 reviews Transformer models, attention mechanisms, and UAV image recognition. Section 3 presents the proposed AeroGRT framework and mathematical formulation. Section 4 reports datasets, implementation settings, ablation studies, comparative experiments, plots, and analysis. Section 5 concludes the paper and discusses future research directions.

## 2. Related Work

### 2.1. Transformer Model

The Transformer architecture introduced multi-head attention as a general mechanism for modeling dependencies among sequence elements [11]. Vision Transformer and DeiT demonstrated that image patches could be treated as visual tokens, enabling global visual recognition with large-scale or data-efficient training [12,13]. DETR formulated object detection as set prediction and replaced hand-designed anchors and NMS-heavy pipelines with Transformer decoding [14]. Hierarchical architectures such as PVT, Swin Transformer, SegFormer, and BiFormer improved dense prediction by combining multi-scale representation, shifted windows, efficient routing, and pyramid design [15,16,17,28].

Despite these advantages, UAV recognition demands additional design constraints. Direct global self-attention may dilute local contour cues and introduce computational overhead. Dynamic snake convolution [29], large-kernel convolution [30], structural re-parameterization [31], and convolutional attention [32] have been introduced to recover geometry, efficiency, and local inductive bias. Therefore, an effective UAV recognition architecture should combine CNN-based local perception with Transformer-based global context modeling.

### 2.2. Attention Mechanism

Attention mechanisms are widely used to model feature interactions in visual tracking. Representative approaches include spatio-temporal Transformer tracking [33], sequence-to-sequence tracking [34], iterative mixed attention [35], shifted-window Transformer tracking [36], and attention-in-attention tracking [37]. UAV-oriented Siamese Transformer pyramids [38] and compact Transformer tracking with correlative masked modeling [39] further extend the range of architectures relevant to this study. A review of Siamese UAV tracking provides broader context for target appearance variation, scale changes, and efficient deployment [40]. These studies motivate attention-based feature interaction for UAV localization and provide relevant tracking architectures for comparison.

Attention mechanisms can benefit from feature complementarity and consistent query–key associations. Feature fusion combines global and local features, cross-axis attention aggregates horizontal and vertical information, and the query-reliability mask weights query–key associations using feature-space consistency. In AeroGRT, these operations are used for global–local scene encoding and multi-level feature fusion for UAV recognition.

### 2.3. UAV Image Monitoring and Recognition

Research relevant to UAV monitoring spans road-distress analysis, traffic-object recognition, and small-object detection. Civil-infrastructure studies investigate crack detection, pavement-distress classification, and benchmark datasets for road-damage analysis [19,20,21,22,23,24,25,26]. Recent UAV detection research includes cooperative mixed-task training [41], lightweight task-aligned detection [42], self-attention-guided global feature fusion [43], and multi-scale Transformer attention for small-object detection [44]. Zunair et al. introduced RSUD20K, a road-scene object detection dataset containing over 20,000 high-resolution images from Bangladesh, with approximately 130,000 bounding-box annotations across 13 object classes [45]. Its crowded traffic scenes, diverse viewpoints, and varying weather conditions are relevant to the background clutter and appearance variability considered in this study. RSUD20K uses a ground-level driving perspective, whereas AeroGRT targets UAV observations involving altitude-related scale and viewpoint changes. It therefore provides complementary context for road-scene perception. These studies motivate the preservation of fine-scale visual evidence together with contextual feature modeling in complex scenes. Table 1 summarizes representative algorithm families and their conceptual relevance to AeroGRT.

## 3. UAV Image Recognition Algorithm Based on Fusion Feature Extraction and Query-Reliability Map Transformation

### 3.1. Interfusion Feature Extraction Network Model

**Figure 2:**
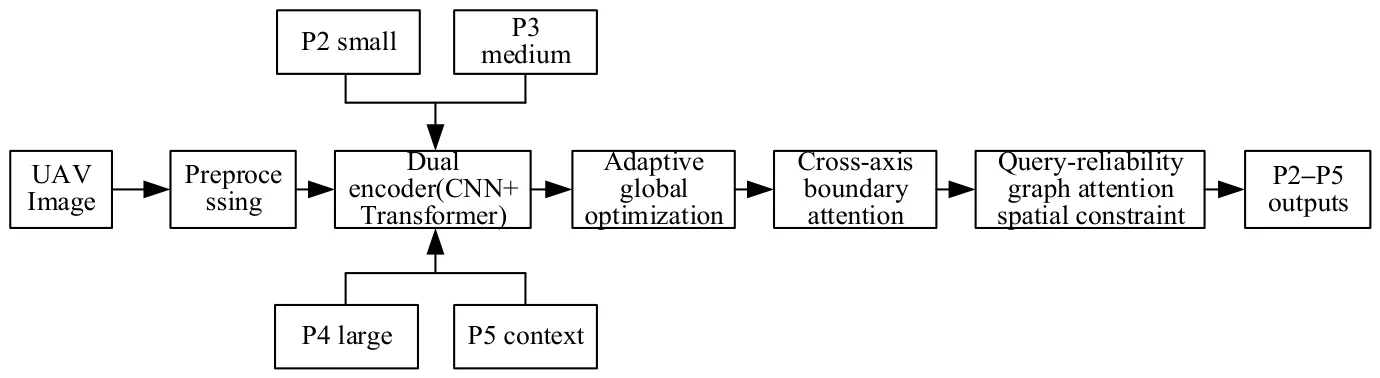
Overall architecture of AeroGRT, including preprocessing, dual-branch feature extraction, global optimization, cross-axis attention, query-reliability graph attention, and scale-altitude routing head.

The overall architecture of AeroGRT is shown in Figure 2. The model receives UAV image frames and optional altitude metadata. The input is first normalized and resized; then a CNN-based local branch extracts fine-grained feature maps, while a Transformer-based global branch models long-range semantic dependencies. The global optimization module forms stage-weighted global representations, and the fusion attention module aligns them with local features at four fusion stages to construct complementary foreground- and background-weighted representations. The resulting features are used by the cross-axis attention module, query-reliability graph module, and scale–altitude routing detection head.

(1) Complementary Feature Extraction Module

**Figure 3:**
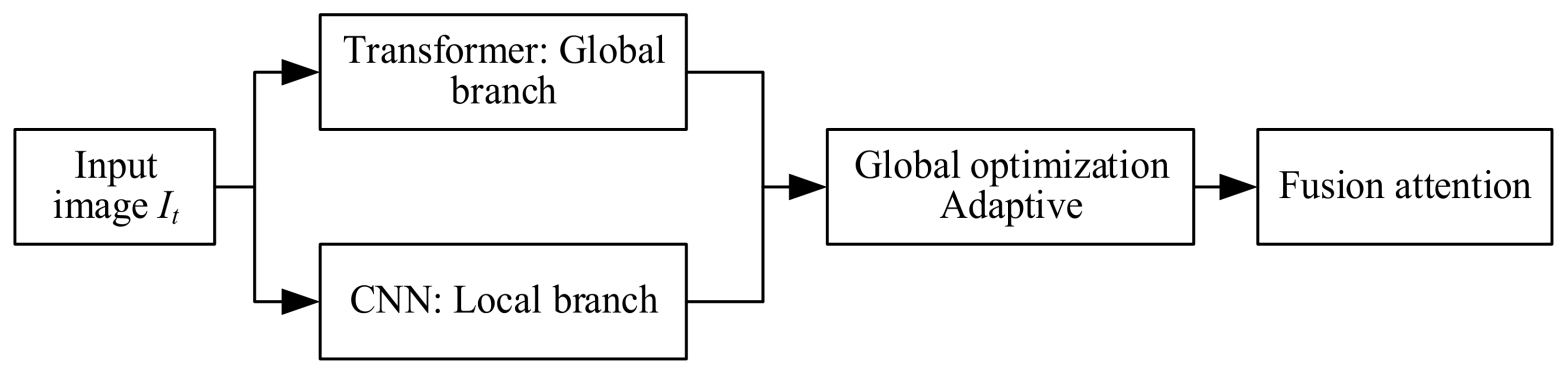
Detailed structure of the interfusion feature extraction network model.

The complementary feature extraction module is shown in Figure 3. Let *I_t_* denote the input UAV image at time *t*. The global branch partitions *I_t_* into non-overlapping patches and embeds them into a token sequence *U*_0_. After *L* Transformer layers, the final token sequence *U_L_* represents scene-level relationships. In parallel, the local branch extracts a hierarchy of convolutional feature maps *F*_1_, *F*_2_, *F*_3_, and *F*_4_, which retain local edges, textures, and small-object details.

Equation (1) defines patch embedding with positional encoding, Equation (2) describes the Transformer updates, Equation (3) constructs the local feature hierarchy, and Equation (4) collects the outputs of the two branches. The final global sequence $U_{L}\in R^{N_{\mathrm{tok}}\times d}$ contains only spatial patch tokens for feature fusion, with no classification token included in this sequence. Here, *N*_tok_ = *h_u_w_u_*, where *h_u_* and *w_u_* denote the height and width of the token grid, respectively, and *d* denotes the token dimension. The local feature at stage *j* is $F_{j}\in R^{c_{j}\times h_{j}\times w_{j}}$, where *c_j_*, *h_j_*, and *w_j_* denote its channel count, height, and width. The positional encoding *E*_pos_ has the same shape as the patch embedding. Throughout this section, Cat denotes concatenation along the channel dimension, and ⊙ denotes element-wise multiplication with broadcasting where specified.

**U**_0_ = PatchEmbed(**I***_t_*) + **E**_pos_ (1)

**U**_*ℓ*_ = 𝒯_*ℓ*_(**U**_*ℓ*−1_), *ℓ* = 1, …, *L* (2)

**F***_j_* = 𝒞*_j_*(**F***_j_*_−1_), *j* = 1, …, 4,  **F**_0_ = **I***_t_* (3)

*Ω* = {**U***_L_*, **F**_1_, **F**_2_, **F**_3_, **F**_4_} (4)

(2) Global Optimization Module

**Figure 4:**
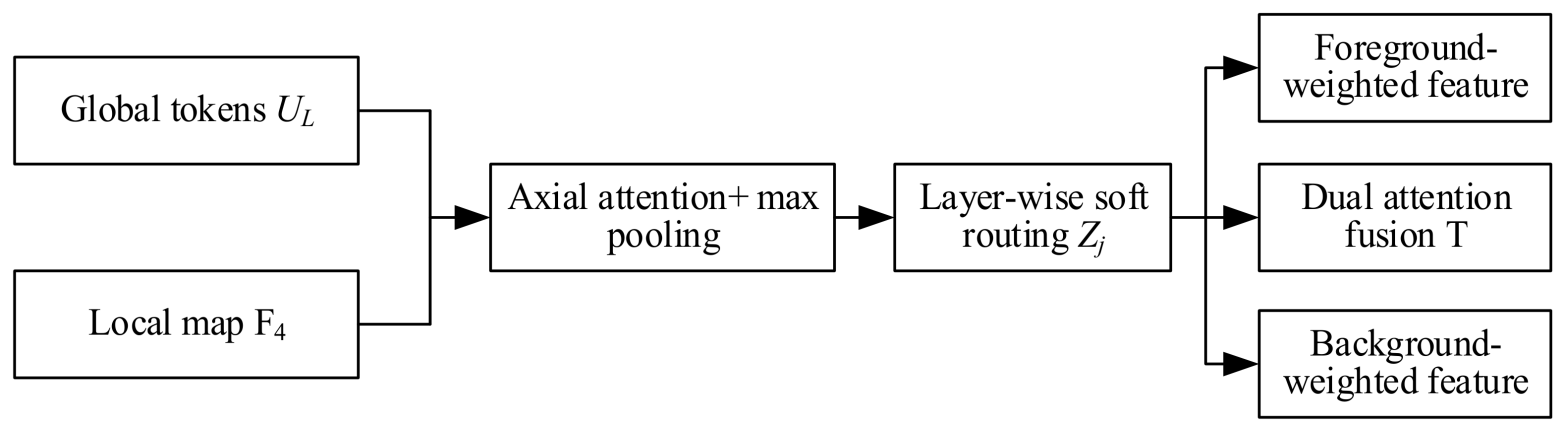
Detailed structure of the global optimization and foreground–background fusion attention module.

The global optimization module provides stage-weighted global representations for the four local–global fusion stages. As shown in Figure 4, AxAttn denotes shape-preserving axial self-attention applied first along the horizontal axis and then along the vertical axis of *F*_4_. Both operations preserve the channel count *c*_4_ and the spatial size *h*_4_ × *w*_4_. The spatial tokens *U_L_* are reshaped into a feature map of size *d* × *h_u_* × *w_u_*, and adaptive max pooling produces an aligned feature $\tilde{U}\in R^{d\times h_{4}\times w_{4}}$. Equation (5) concatenates AxAttn(*F*_4_) and $\tilde{U}$ along the channel dimension, giving $Z_{\mathrm{gl}}\in R^{c_{\mathrm{gl}}\times h_{4}\times w_{4}}$, where *c*_gl_ = *c*_4_ + *d*. In Equation (6), each Gate*_j_* is an independent 1 × 1 convolution mapping *c*_gl_ channels to a single-channel score map. Global average pooling over the spatial dimensions yields a scalar score *e_j_*, and a softmax over the four stages produces weights satisfying $\sum_{j=1}^{4}\alpha_{j}=1$. Equation (7) scales the shared global representation for each fusion stage. The subsequent stage-dependent alignment operator ℛ*_j_* maps *Z_j_* to the shape of the corresponding local feature.

(5) $$Z_{\mathrm{gl}}=\mathrm{Cat}\;\left[{\mathrm{AxAttn}}_{\mathrm{ver}}({\mathrm{AxAttn}}_{\mathrm{hor}}(F_{4})),{\mathrm{AdaptiveMaxPool}}_{h_{4},w_{4}}({\mathrm{Reshape}}_{h_{u},w_{u}}(U_{L}))\right]$$

(6) $$\alpha_{j}=\frac{\exp(\mathrm{GAP}({\mathrm{Gate}}_{j}(Z_{\mathrm{gl}})))}{\sum_{r=1}^{4}\exp(e_{r})},j=1,\ldots ,4$$

***Z****_j_* = *α_j_* ⊙ *Z*_gl_, *j* = 1, …, 4 (7)

Each axial operation applies self-attention independently to the sequences along its specified spatial axis and projects the output back to *c*_4_ channels. The adaptive max-pooling output size is fixed to (*h*_4_, *w*_4_), and all gating convolutions use stride 1.

(3) Fusion Attention Module

The fusion attention module forms complementary foreground- and background-weighted representations at each fusion stage. First, the global representation *Z_j_* is bilinearly resized to *h_j_* × *w_j_* and projected by a stage-dependent 1 × 1 convolution from *c*_gl_ channels to *c_j_* channels. This operation is denoted by ${\tilde{Z}}_{j}=R_{j}(Z_{j})$, giving ${\tilde{Z}}_{j}\in R^{c_{j}\times h_{j}\times w_{j}}$, which has the same shape as *F_j_*. Equation (8) concatenates these features along the channel dimension and uses a 1 × 1 convolution with one output channel, followed by a sigmoid, to produce the spatial foreground mask $B_{j}\in [0,1{]}^{1\times h_{j}\times w_{j}}$. In Equations (9) and (10), *B_j_* and its complement 1 − *B_j_* are broadcast over the feature channels to form foreground- and background-weighted features, respectively. Equations (11) and (12) aggregate the two branches from stage 4 to stage 1, with the recursion evaluated in the order *j* = 3, 2, 1. The operator 𝒰*_j_* first bilinearly resizes a feature from stage *j* + 1 to *h_j_* × *w_j_* and then applies a 1 × 1 convolution from *c_j_*_+1_ channels to *c_j_* channels. Its parameters are shared between the foreground and background branches at the same stage but differ across stages. All 1 × 1 convolutions use stride 1, and all bilinear interpolation uses align_corners = False. Finally, Equation (13) transforms the difference between the aggregated foreground and background features into the output representation *T*.

(8) $${\tilde{Z}}_{j}=R_{j}\left(Z_{j}\right),B_{j}=\sigma \left({\mathrm{Conv}}_{1\times 1}\left(\mathrm{Cat}\left[F_{j},R_{j}\left(Z_{j}\right)\right]\right)\right),j=1,2,3\ldots 4$$

(9) $$F_{j}^{\mathrm{fg}}=B_{j}⊙F_{j}+B_{j}⊙{\tilde{Z}}_{j}$$

(10) $$F_{j}^{\mathrm{bg}}=(1-B_{j})⊙F_{j}+(1-B_{j})⊙{\tilde{Z}}_{j}$$

(11) $$G_{4}^{\mathrm{fg}}=F_{4}^{\mathrm{fg}},\;\;G_{j}^{\mathrm{fg}}=F_{j}^{\mathrm{fg}}+U_{j}(G_{j+1}^{\mathrm{fg}}),\;j=1,2,3$$

(12) $$G_{4}^{\mathrm{bg}}=F_{4}^{\mathrm{bg}},\;\;G_{j}^{\mathrm{bg}}=F_{j}^{\mathrm{bg}}+U_{j}(G_{j+1}^{\mathrm{bg}}),\;j=1,2,3$$

(13) $$T=\mathrm{PReLU}[{\mathrm{Conv}}_{3\times 3}(|G_{1}^{\mathrm{fg}}-G_{1}^{\mathrm{bg}}|)]$$

### 3.2. UAV Recognition Algorithm Based on Cross-Axis Transformer Model

The AeroGRT cross-axis Transformer model includes a cross-axis attention mechanism, a query-reliability mask attention mechanism, a prediction head, and a training function. The AeroGRT model is more suitable for UAV detection and monitoring. Template–search feature interaction can be generalized to interactions between scene labeling, multi-scale feature maps, and detection queries.

(1) Cross-Axis Attention Mechanism

**Figure 5:**
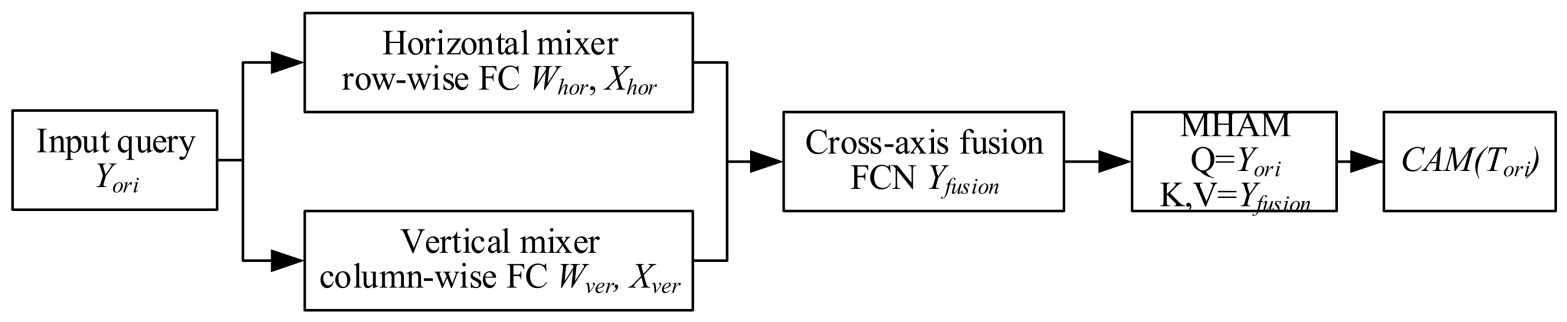
Specific details of the cross-axis attention mechanism for UAV image recognition.

The cross-axis attention mechanism is shown in Figure 5. Equation (14) computes scaled dot-product attention for each head, and Equation (15) concatenates the head outputs. Here, *n*_head_ is the number of attention heads, *d*_head_ is the feature dimension of each head, and *r* indexes the heads. Equations (16) and (17) denote the attention and feed-forward substeps of an encoder layer, respectively, with ${\tilde{Z}}_{\mathrm{enc}}^{\left(\ell \right)}$ denoting the intermediate representation. The directional transformation block uses a fixed working grid of size *h_y_* × *w_y_*. Before directional transformation, an incoming feature with a different grid is bilinearly resized to this working grid using align_corners = False. The resulting input is denoted by $Y_{\mathrm{ori}}\in R^{c_{y}\times h_{y}\times w_{y}}$, where *c_y_* = *n*_head_*d*_head_. Equation (18) applies horizontal and vertical transformations separately to each channel, with $W_{\mathrm{hor}}\in R^{w_{y}\times w_{y}}$ and $W_{\mathrm{ver}}\in R^{h_{y}\times h_{y}}$. Equation (19) concatenates the outputs along the channel dimension and uses a 1 × 1 convolution, denoted by FCN, to map 2*c_y_* channels back to *c_y_* channels while preserving the working grid. Equation (20) flattens the spatial dimensions into token sequences, uses the original sequence as the query and the fused sequence as the key–value source, and reshapes the attention output back to the working grid.

(14) $$O_{r}^{\mathrm{head}}=\mathrm{Softmax}(\frac{Q_{r}K_{r}^{⊤}}{\sqrt{d_{\mathrm{head}}}})V_{r},\;r=1,\ldots ,n_{\mathrm{head}}$$

(15) $$\mathrm{MHAM}(Q,K,V)=\mathrm{Concat}(O_{1}^{\mathrm{head}},\ldots ,O_{n_{\mathrm{head}}}^{\mathrm{head}})W^{O}$$

(16) $${\tilde{Z}}_{\mathrm{enc}}^{\left(\ell \right)}=\mathrm{LN}[Z_{\mathrm{enc}}^{\left(\ell -1\right)}+\mathrm{MHAM}(Z_{\mathrm{enc}}^{\left(\ell -1\right)},Z_{\mathrm{enc}}^{\left(\ell -1\right)},Z_{\mathrm{enc}}^{\left(\ell -1\right)})]$$

(17) $$Z_{\mathrm{enc}}^{\left(\ell \right)}=\mathrm{LN}[{\tilde{Z}}_{\mathrm{enc}}^{\left(\ell \right)}+\mathrm{FFN}({\tilde{Z}}_{\mathrm{enc}}^{\left(\ell \right)})]$$

**X***_hor_* = **W***_hor_***Y**_ori_,  **X***_ver_* = **Y**_ori_**W***_ver_* (18)

**Y**_fusion_ = FCN (Cat[**X***_hor_*,**X***_ver_*]) (19)

CAM(**Y**_ori_) = MHAM(**Y**_ori_**W***^Q^*,**Y**_fusion_**W***^K^*,**Y**_fusion_**W***^V^*) (20)

(2) Query-Reliability Mask Attention

**Figure 6:**
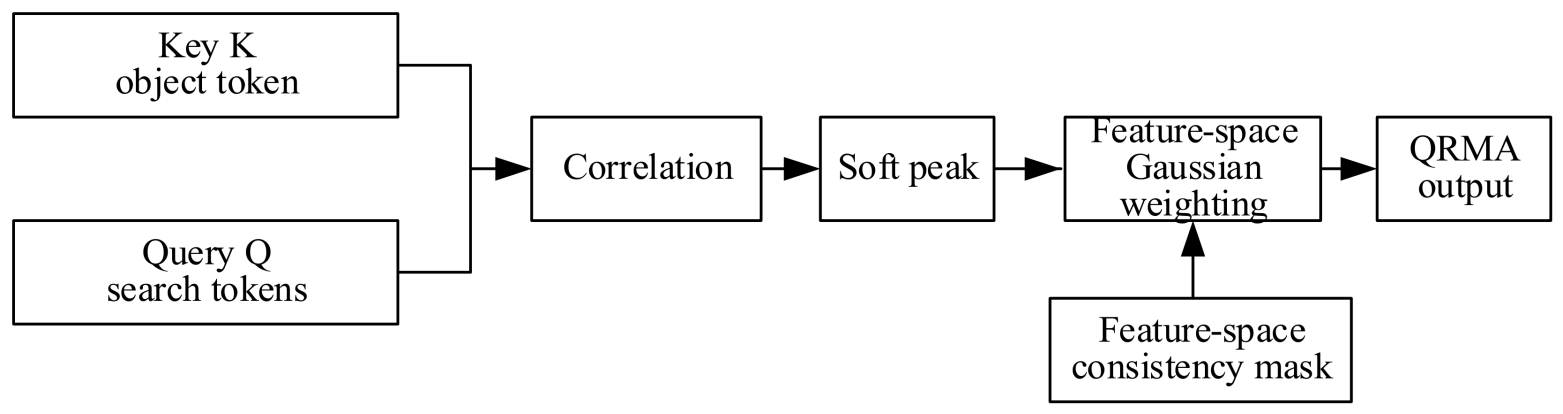
Flowchart of the query-reliability mask attention mechanism and its feature-space consistency mask.

The query-reliability graph attention module is shown in Figure 6. In complex UAV scenes, foreground and background tokens can have similar features, resulting in ambiguous query–key associations. Equations (21)–(24) describe the computation for one attention head. Let $q_{i},k_{k}\in R^{d_{\mathrm{head}}}$ denote the query and key feature vectors, respectively, where *i* = 1, …, *N_q_* and *k* = 1, …, *N_k_*. Equation (21) defines their similarity scores and the strongest unperturbed query correspondence $i_{k}^{*}$ for each key. The index $i_{k}^{*}$ explains the hard correspondence, while differentiable matching uses the distribution in Equation (22). The correspondence probabilities *ϕ_ki_* are obtained by normalizing the similarity scores over queries for each key. Equation (22) applies a Gumbel–Softmax relaxation and computes the weighted query feature ***μ****_k_*. During training, the noise variables *ξ_i_* are independently sampled from the standard Gumbel distribution and shared across keys; during inference, *ξ_i_* = 0. The temperature *τ* > 0 controls the softness of the correspondence distribution. Equation (23) constructs a feature-space consistency mask based on the distance between each query feature and ***μ****_k_*, where *λ* > 0 controls the mask bandwidth. Equation (24) incorporates this mask into query-to-key attention before aggregating the value vectors **v***_k_*. The correspondence distribution in Equation (22) is normalized over queries for each key, whereas the attention weights in Equation (24) are normalized over keys for each query. This formulation favors feature-consistent query–key associations without assigning a spatial interpretation to feature distances.

(21) $$i_{k}^{*}=\underset{1\leq i\leq N_{q}}{\mathrm{argmax}}\;a_{\mathrm{kq}},\;\;a_{\mathrm{ki}}=\frac{q_{i}^{⊤}k_{k}}{\sqrt{d_{\mathrm{head}}}}$$

(22) $$\pi_{\mathrm{ki}}=\frac{\exp[(a_{\mathrm{ki}}+\xi_{i})/\tau ]}{\sum_{r=1}^{N_{q}}\exp[(a_{\mathrm{kr}}+\xi_{r})/\tau ]},\;\;\mu_{k}=\sum_{i=1}^{N_{q}}\pi_{\mathrm{ki}}q_{i}$$

(23) $$N_{\mathrm{ki}}=\exp(-\frac{{\left\|q_{i}-\mu_{k}\right\|}_{2}^{2}}{\lambda^{2}})$$

(24) $$w_{\mathrm{ik}}=\frac{\exp(a_{\mathrm{ki}})N_{\mathrm{ki}}}{\sum_{r=1}^{N_{k}}\exp(a_{\mathrm{ri}})N_{\mathrm{ri}}},o_{i}=\sum_{k=1}^{N_{k}}w_{\mathrm{ik}}v_{k}$$

(3) Scale–Altitude Routing Detection Head

**Figure 7:**
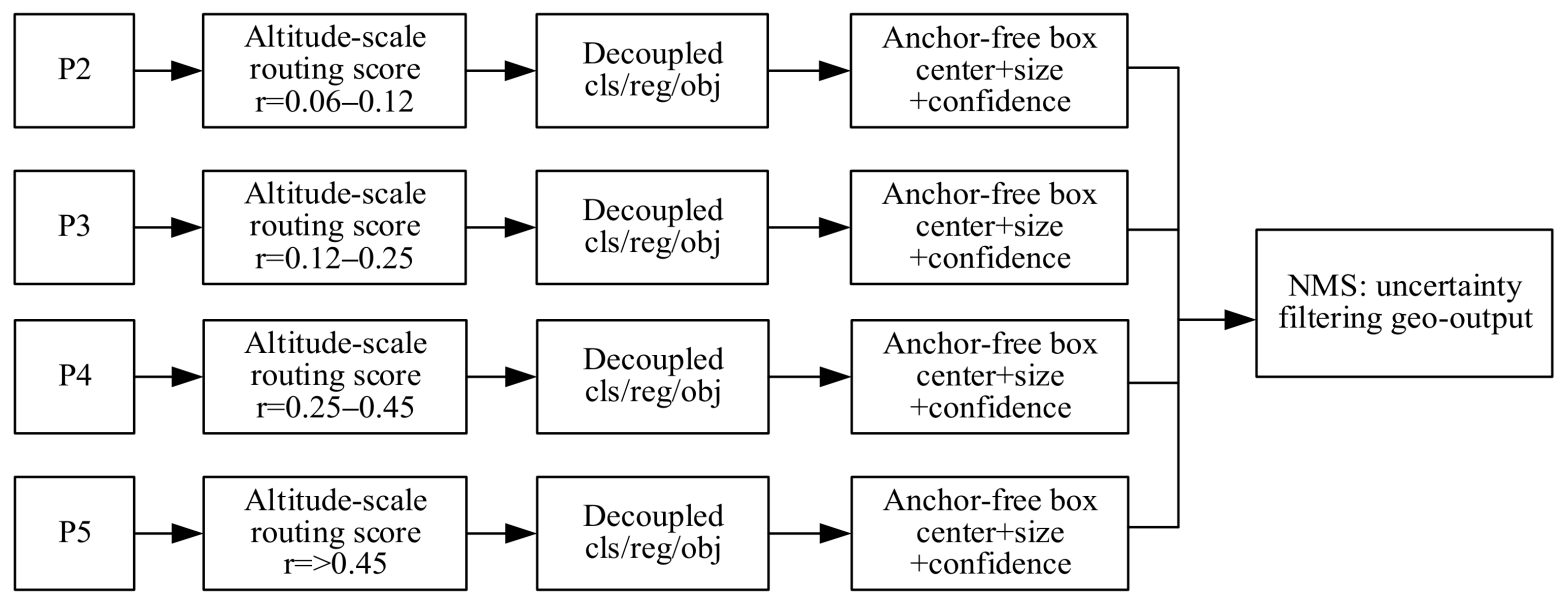
Scale–altitude routing detection head with decoupled classification, regression, and objectness branches.

The scale–altitude routing detection head is shown in Figure 7. It estimates image-level priors over pyramid levels *P*_2_–*P*_5_. Equation (25) globally pools each pyramid feature to obtain a scale-specific descriptor. Equation (26) concatenates these descriptors with normalized altitude metadata and an altitude-availability flag, and a two-layer MLP predicts four routing weights using softmax. Scale-related information is inferred from the pyramid features, and no ground-truth bounding-box dimensions are used as routing inputs during either training or inference. Each pyramid level retains decoupled classification, regression, and objectness branches. Equation (27) applies the routing weight once to the joint detection confidence, while retaining the decoded bounding-box coordinates without scaling. Candidates from all four levels are concatenated, filtered by confidence, and processed using class-wise non-maximum suppression. The routing network is trained jointly with the detection branches through the routed classification confidence.

(25) $$P_{\ell }\in R^{c_{\ell }^{P}\times h_{\ell }^{P}\times w_{\ell }^{P}},u_{\ell }=\mathrm{GAP}(P_{\ell })\in R^{c_{\ell }^{P}},\ell =2,3,4,5$$

(26) $$m_{t}=\{\begin{array}{ll} 1, & \mathrm{altitude metadata are available},\\ 0, & \mathrm{otherwise}, \end{array}{\tilde{a}}_{t}^{\mathrm{alt}}=\{\begin{array}{ll} \frac{a_{t}^{\mathrm{alt}}-\mu_{a}}{\sigma_{a}+\varepsilon }, & m_{t}=1,\\ 0, & m_{t}=0. \end{array}$$

(27) $$\begin{array}{ll} r_{\ell \mathrm{uc}} & =s_{t,\ell }o_{\ell u}p_{\ell \mathrm{uc}},\\ Y_{t} & ={\mathrm{NMS}}_{\delta_{\mathrm{nms}}}({\mathrm{Concat}}_{\ell =2}^{5}\{({\hat{b}}_{\ell u},c,r_{\ell \mathrm{uc}}):r_{\ell \mathrm{uc}}\geq \delta_{\mathrm{score}}\}). \end{array}$$

Here, *u* indexes candidate detections, *c* = 1, …, *n*_cls_ indexes object classes, *o*_*ℓu*_ is the objectness probability, and *p*_*ℓuc*_ is the conditional class probability. The decoded boxes ${\hat{b}}_{\ell u}$ from all pyramid levels are expressed in the same image coordinate system. The thresholds *δ*_score_ and *δ*_nms_ control confidence filtering and class-wise NMS, respectively. Routing weights modify detection confidence only; they do not multiply bounding-box coordinates. During training, label assignment and Equation (28) use dense predictions before confidence filtering and NMS. Confidence filtering and class-wise NMS are applied only during inference.

(4) Training Function

AeroGRT uses a multi-task objective consisting of classification loss, box regression loss, and query-consistency regularization. Let *m* index the valid prediction–class pairs after label assignment, let *y_m_* ∈ {0, 1} denote the corresponding binary label, and let *r_m_* denote the routed joint confidence defined in Equation (27). The probability assigned to the observed label is $p_{m}^{*}=y_{m}r_{m}+(1-y_{m})(1-r_{m})$. Equation (28) applies focal loss to this probability, where $\omega_{y_{m}}$ is the class-balancing weight and *γ* ≥ 0 is the focusing parameter. Equation (29) computes box regression loss over the *N*_pos_ positive matches, where ${\hat{b}}_{u}$ and $b_{u}^{\mathrm{gt}}$ denote predicted and ground-truth boxes, respectively, and *β*_1_,*β*_2_ ≥ 0 weight the L1 and GIoU terms. The box loss is zero when there are no positive matches. Equation (30) regularizes the agreement between the normalized feature-space consistency mask and the unmasked key-to-query correspondence distribution. For each key *k*, both distributions are normalized over queries, with ${\bar{N}}_{\mathrm{ki}}=N_{\mathrm{ki}}/\sum_{r=1}^{N_{q}}N_{\mathrm{kr}}$ and ${\bar{A}}_{\mathrm{ki}}=\phi_{\mathrm{ki}}$. They differ from the query-to-key attention weights *w_ik_* in Equation (24). The constant 0 < *ε* < 1 provides numerical stability in the logarithms. Equation (31) combines the three losses, with *β*_3_ ≥ 0 controlling the contribution of query-consistency regularization.

(28) $$p_{m}^{⋆}={y_{m}r_{m}+\left(1-y_{m}\right)\left(1-r_{m}\right),L}_{\mathrm{cls}}=-\frac{1}{N_{\mathrm{samp}}}\sum_{m=1}^{N_{\mathrm{samp}}}\omega_{y_{m}}(1-p_{m}^{⋆}{)}^{\gamma }\log(\max(p_{m}^{⋆},\varepsilon ))$$

(29) $$L_{\mathrm{box}}=\frac{1}{N_{\mathrm{pos}}}\sum_{u=1}^{N_{\mathrm{pos}}}[\beta_{1}\|{\hat{b}}_{u}-b_{u}^{\mathrm{gt}}{\|}_{1}+\beta_{2}(1-\mathrm{GIoU}({\hat{b}}_{u},b_{u}^{\mathrm{gt}}))]$$

(30) $${\bar{N}}_{\mathrm{ki}}=\frac{N_{\mathrm{ki}}}{\sum_{r=1}^{N_{q}}N_{\mathrm{kr}}},{\bar{A}}_{\mathrm{ki}}=\phi_{\mathrm{ki}}=\frac{\exp(a_{\mathrm{ki}})}{\sum_{r=1}^{N_{q}}\exp(a_{\mathrm{kr}})}L_{\mathrm{rel}}=\frac{1}{N_{k}}\sum_{k=1}^{N_{k}}\sum_{i=1}^{N_{q}}{\bar{N}}_{\mathrm{ki}}\log(\frac{{\bar{N}}_{\mathrm{ki}}+\varepsilon }{{\bar{A}}_{\mathrm{ki}}+\varepsilon })$$

ℒ_total_ = ℒ_cls_ + ℒ_box_ + *β*_3_ℒ_rel_ (31)

The routing MLP receives gradients through the routed confidence in Equation (28), while box regression uses decoded boxes before confidence routing. The class-balancing weights satisfy ***ω*_0_**,***ω*_1_** ≥ **0**. The classification loss is zero when there are no valid prediction–class pairs. The same routing computation is used during training and inference, with confidence filtering and NMS restricted to inference.

## 4. Experiments and Analysis

### 4.1. Environment Setting and Datasets

Table 2 summarizes the benchmark datasets considered for evaluating UAV recognition, tracking, and monitoring capabilities. These datasets cover single-object tracking, large-scale visual tracking, UAV-based object detection, small-object detection, and road defect recognition tasks. Among them, GOT-10k, TrackingNet, VisDrone2019, and TinyPerson are adopted for quantitative evaluation, while other datasets are introduced as representative benchmarks related to UAV perception and monitoring applications.

The GOT-10k dataset is adopted for single-object tracking evaluation. It contains 10,000 training sequences and 180 test sequences, covering more than 560 object categories and diverse motion patterns. Different from conventional tracking benchmarks, GOT-10k follows a strict one-shot evaluation protocol, where object categories in the test set do not overlap with those in the training set. Therefore, it provides a challenging benchmark for evaluating the generalization ability of tracking models under unseen object categories and complex visual variations.

The TrackingNet dataset is further used to evaluate large-scale tracking performance in real-world scenarios. It contains 30,132 training sequences and 511 test sequences collected from YouTube videos, with approximately 14 million annotated bounding boxes. It emphasizes tracking robustness under diverse appearance changes and complex environmental conditions.

For UAV-based object detection evaluation, the VisDrone2019 and TinyPerson datasets are selected. VisDrone2019 contains images captured from multiple urban environments with dense small objects, while TinyPerson focuses on tiny pedestrian detection from high-altitude views. These datasets provide challenging scenarios for evaluating UAV perception under scale variation, background interference, and low-resolution targets.

**Figure 8:**
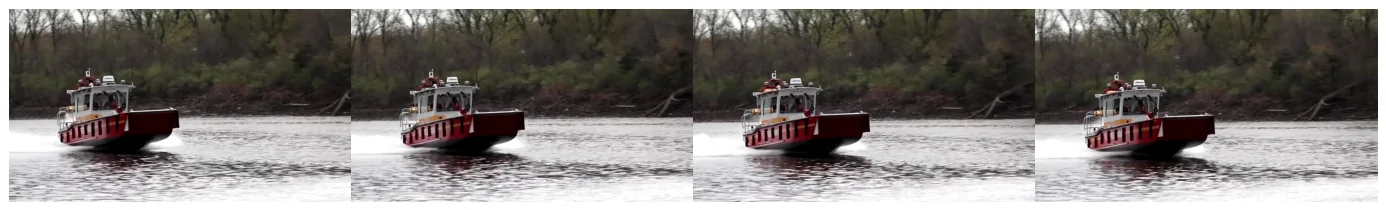
Representative tracking samples from the GOT-10k dataset.

To provide a clearer understanding of the tracking task, representative samples from the GOT-10k dataset are presented in Figure 8. The examples illustrate target appearance variation, scale changes, background interference, and motion deformation.

The detailed information of all benchmark datasets is summarized in Table 2.

**Figure 9:**
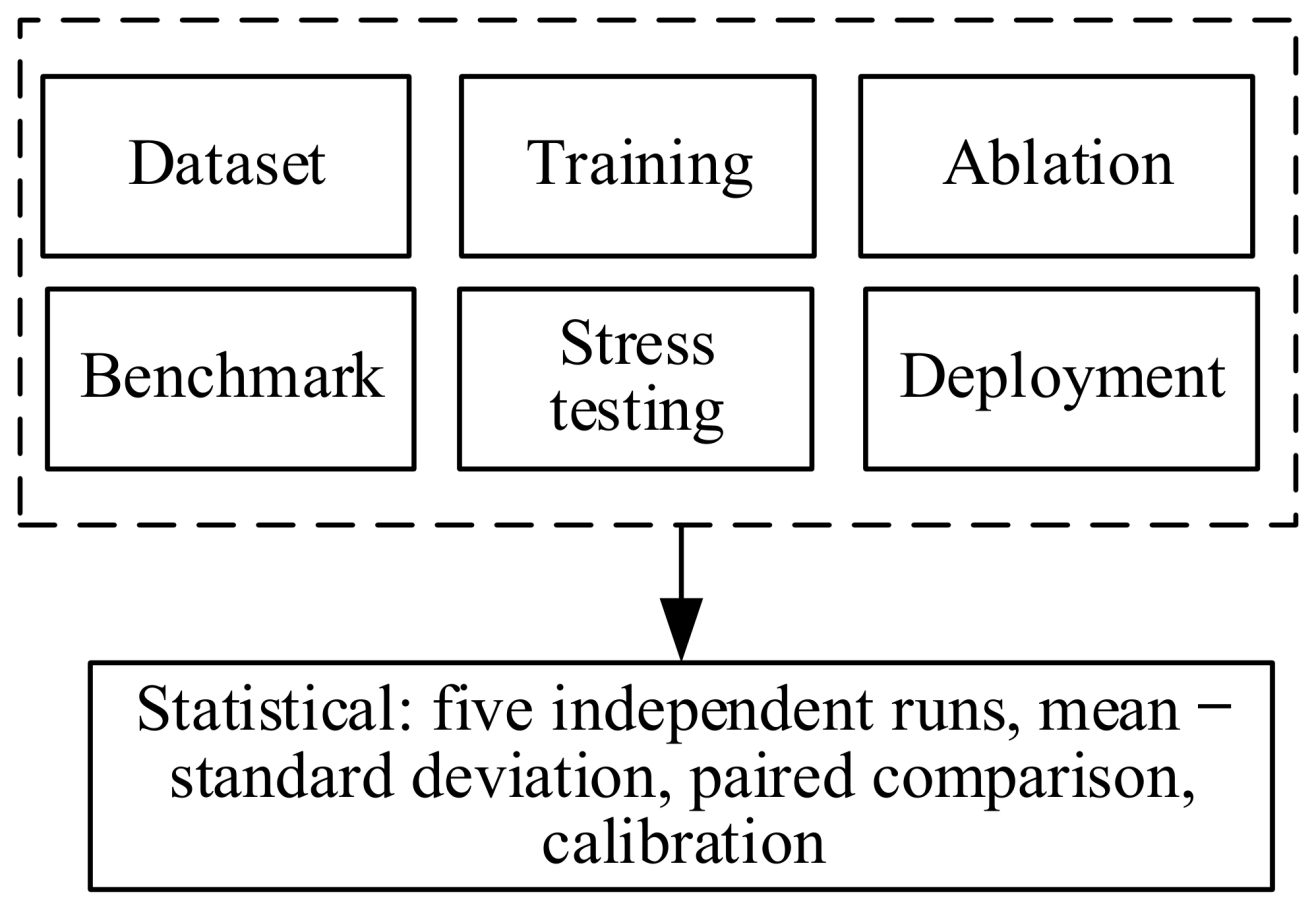
Pipeline for benchmark evaluation and planned edge deployment.

For a complete AeroGRT implementation, the default training setting is PyTorch 2.14.1 with AdamW or SGD, an initial learning rate of 1 × 10^−4^ and a weight decay of 1 × 10^−4^, 300 training epochs, a batch size of 8, mosaic and mixup augmentation for detection datasets, random shift and blur augmentation for tracking datasets, and multi-scale training for UAV object detection. We plan to explore ONNX model export and TensorRT inference optimization, followed by measurements of end-to-end latency, frame rate, power consumption, and memory usage on Jetson-class embedded devices to assess deployment feasibility. The evaluation pipeline is shown in Figure 9. Equation (32) defines precision, Equation (33) defines recall, Equation (34) defines class-wise AP and the mean AP over all classes, Equation (35) gives the harmonic F1 measure, and Equation (36) reports inference throughput in frames per second.

(32) $$\mathrm{Precision}=\frac{\mathrm{TP}}{\mathrm{TP}+\mathrm{FP}}$$

(33) $$\mathrm{Recall}=\frac{\mathrm{TP}}{\mathrm{TP}+\mathrm{FN}}$$

(34) $${\mathrm{AP}}_{c}=\int_{0}^{1}P_{c}\left(R\right)\;dR,\;\;\mathrm{mAP}=\frac{1}{C}\sum_{c=1}^{C}{\mathrm{AP}}_{c}$$

(35) $$F_{1}=2\;\frac{\mathrm{Precision}\cdot \mathrm{Recall}}{\mathrm{Precision}+\mathrm{Recall}}$$

(36) $$\mathrm{FPS}=\frac{N_{\mathrm{frames}}}{T_{\mathrm{inference}}}$$

### 4.2. Ablation Experiments

**Figure 10:**
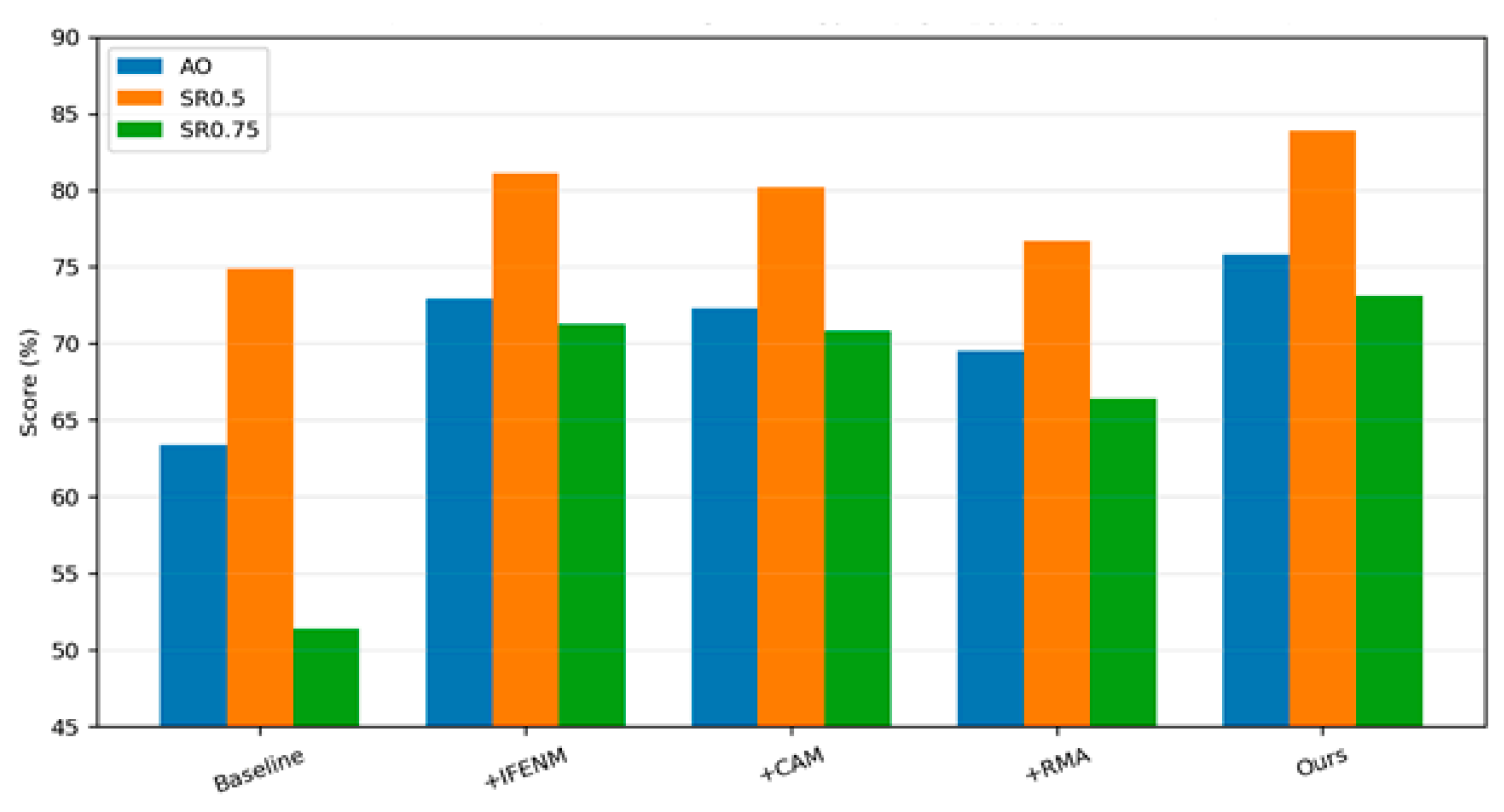
Ablation experiment results on the Got-10k dataset.

Table 3 lists the ablation results on GOT-10k. IFENM, CAM, and RMA denote the interfusion feature extraction module, cross-axis attention module, and query-reliability mask attention module, respectively. The baseline has an AO of 63.4%. The individual IFENM, CAM, and RMA variants have AO values of 72.9%, 72.3%, and 69.5%, respectively. The IFENM + CAM, CAM + RMA, and IFENM + RMA combinations have AO values of 73.4%, 72.8%, and 74.1%, respectively. The complete combination has an AO of 75.8%, an SR0.5 of 83.9%, and an SR0.75 of 73.1%. Figure 10 visualizes the ablation results.

**Figure 11:**
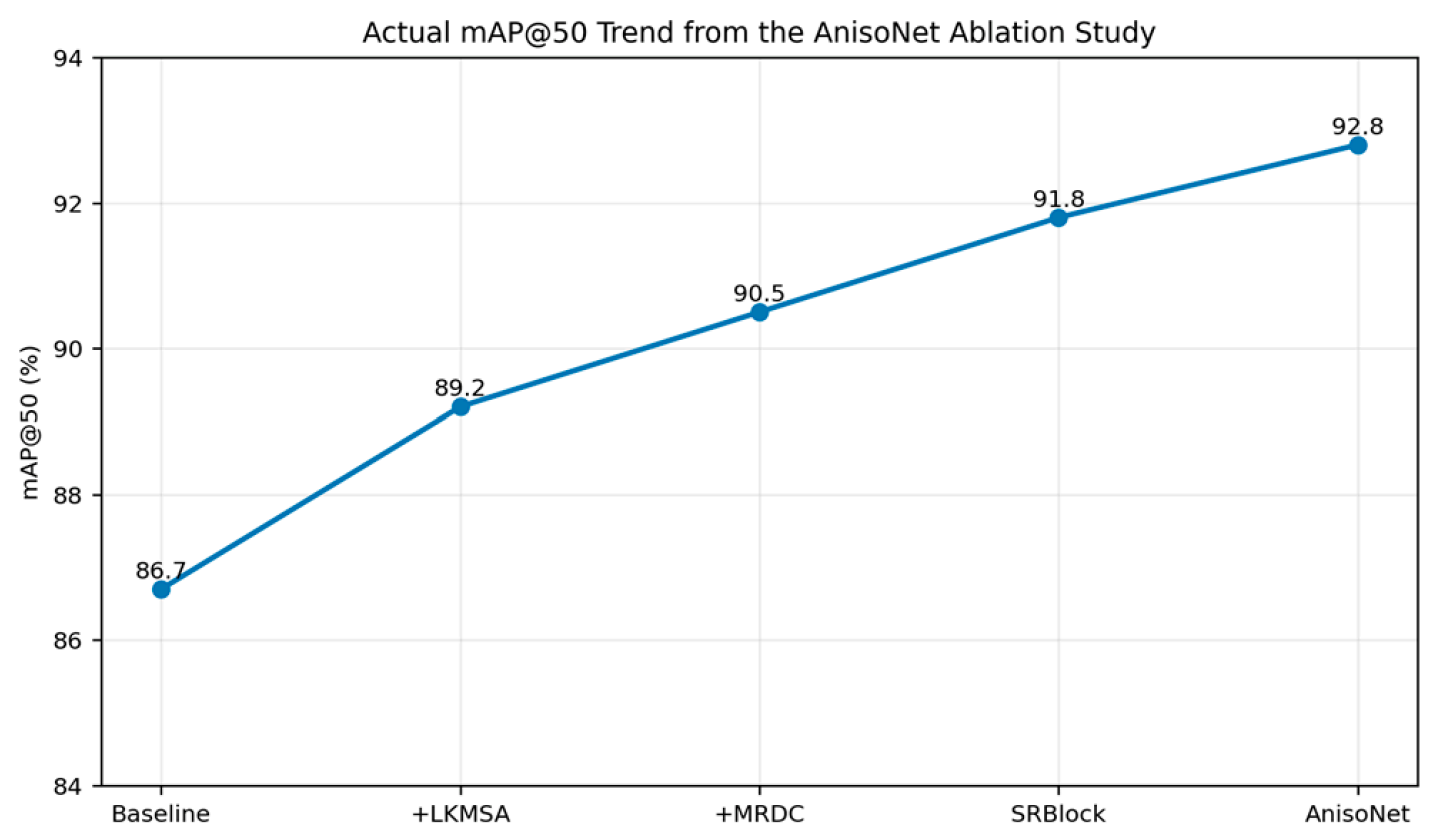
Plot of mAP@50 trend from the AnisoNet ablation study.

Table 4 summarizes the ablation trend reported by AnisoNet for geometry-aware recognition. The baseline reaches 86.7% mAP@50. Adding LKMSA increases mAP@50 by 2.5 percentage points while reducing FPS only moderately. MRDC raises mAP@50 to 90.5%, SRBlock reaches 91.8%, and the complete AnisoNet architecture reaches 92.8%. Figure 11 visualizes this mAP@50 trend. These results motivate the use of geometry-aware feature modeling in AeroGRT.

The performance results are reported as mean ± standard deviation (mean ± SD). A smaller standard deviation indicates that the model achieves more stable performance across different training runs. Furthermore, a paired two-tailed Student’s *t*-test was performed between the baseline Transformer model and AeroGRT to determine whether the observed improvements were statistically significant rather than caused by random fluctuations. The statistical comparison results on the GOT-10k dataset are presented in Table 5.

Compared with the baseline, AeroGRT improves AO, SR0.5, and SR0.75 by 12.3, 9.1, and 21.5 percentage points, respectively. The paired two-tailed Student’s *t*-test indicates statistically significant differences across all three metrics (*p* < 0.001), providing statistical evidence of improved performance across repeated training runs.

### 4.3. Performance Evaluation of Traffic Target Tracking and UAV Recognition

(1) Experimental Results on the Got-10k Dataset

Table 6 presents the comparative tracking results on GOT-10k. The AeroGRT row reports an AO of 75.8%, an SR0.5 of 83.9%, and an SR0.75 of 73.1%, corresponding to gains of 1.0, 2.0, and 0.9 percentage points over the listed SeqTrack results. Its reported inference speed is 60.9 FPS, compared with 68.6 FPS for SimTrack. The table compares tracking accuracy and inference speed across the listed methods

(2) Experiment Results on the TrackingNet Dataset

**Figure 12:**
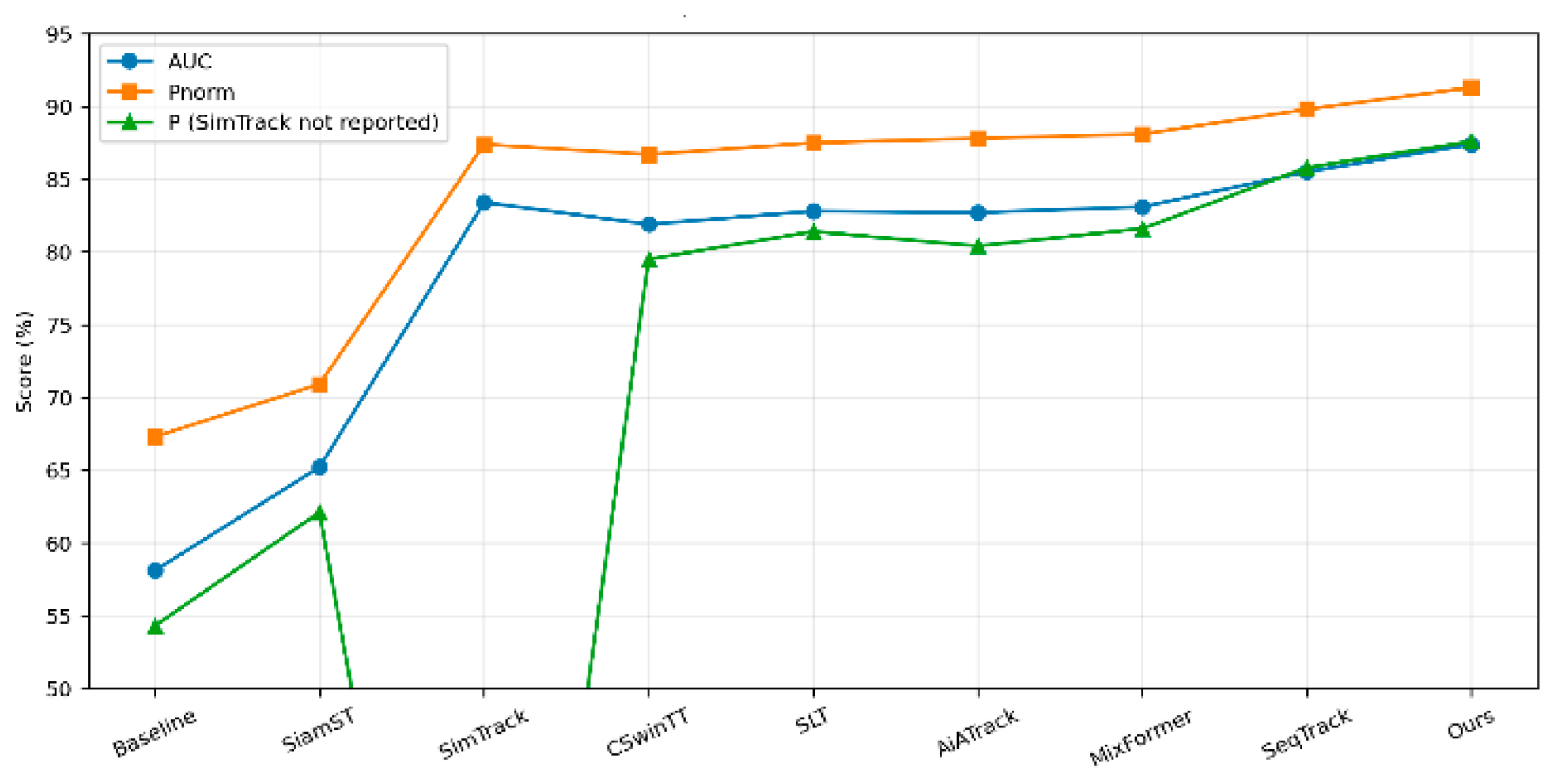
Comparative tracking results on TrackingNet.

Table 7 reports the TrackingNet comparative results. The AeroGRT method achieves AUC of 87.4%, normalized precision of 91.3%, and precision of 87.6%. Compared with SeqTrack, the improvements are 1.9, 1.5, and 1.8 percentage points. Figure 12 plots these results and shows that the method improves both overlap-based and precision-based measures. These results demonstrate AeroGRT’s improved overall tracking performance on TrackingNet.

(3) Small-Object Detection Results on VisDrone and TinyPerson

Table 8 presents AeroGRT’s small-object detection improvements on VisDrone and TinyPerson. On VisDrone, AeroGRT improves mAP50 and mAP50–95 by 2.8 and 1.4 percentage points, respectively, over YOLOv8n + P2 + P3. On TinyPerson, the corresponding improvements over YOLOv8n + P2 + P3 + P4 are 1.1 and 0.2 percentage points. Compared with YOLOv10n + P2 + P3 + P4 + P5 on VisDrone, AeroGRT improves the two metrics by 3.4 and 1.4 percentage points, respectively.

The experimental results reveal that different components of AeroGRT contribute to performance improvement from complementary perspectives. In the AeroGRT tracking experiments, inter-fusion feature extraction provides the largest individual improvement, indicating that global–local feature interaction is beneficial for handling appearance variation and background interference. Cross-attention further enhances localization accuracy by preserving boundary and contour information, while relevance mask attention reduces unreliable query–key associations.

In addition, the geometry-aware mechanisms introduced from AnisoNet provide useful guidance for UAV recognition tasks involving slender, weak-texture, and low-contrast targets. The combination of global–local feature interaction and geometry-adaptive routing enables AeroGRT to better handle challenging UAV scenarios, including high-altitude views, cluttered urban environments, and infrastructure monitoring scenes.

However, the current evaluation mainly focuses on benchmark performance. Further work will address calibration evaluation, edge deployment latency, power consumption, and code availability.

### 4.4. Visualization Experiments

**Figure 13:**
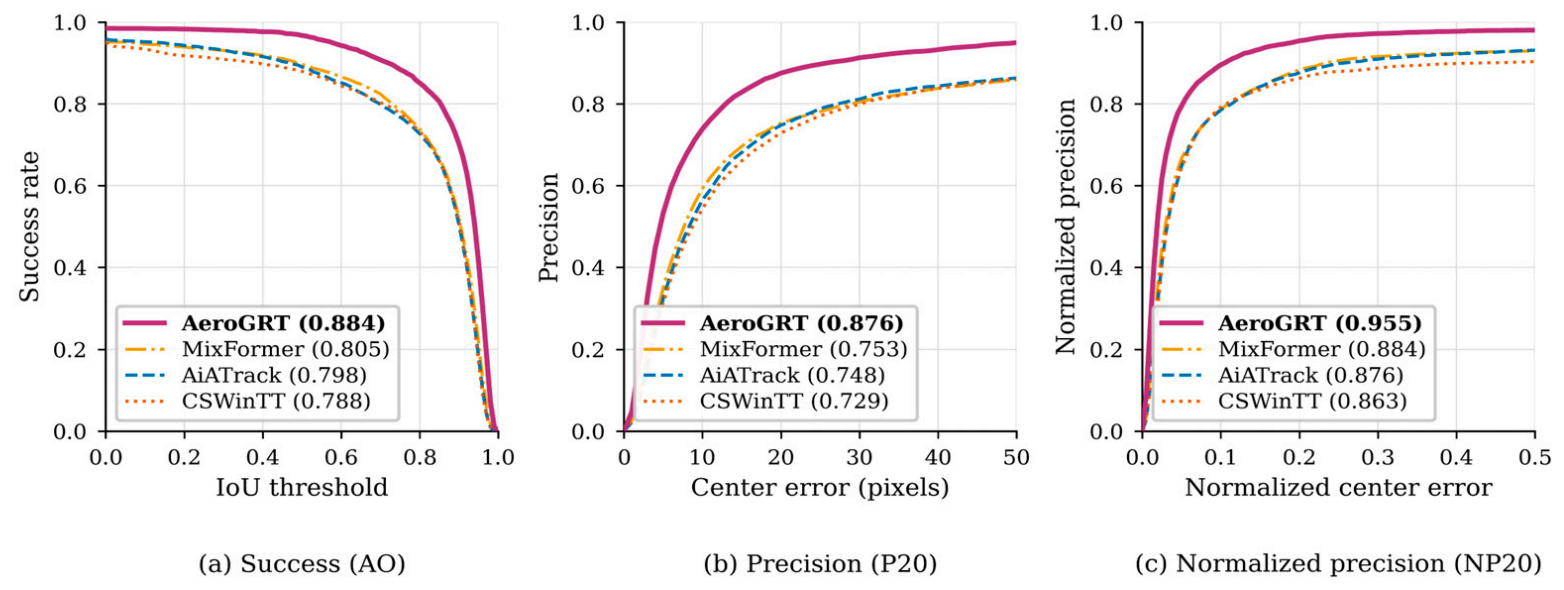
Overall performance. (**a**) Success plot, (**b**) center location precision plot, (**c**) normalized center location precision plot. All curves are calculated per sequence and equally averaged across 50 sequences.

**Figure 14:**
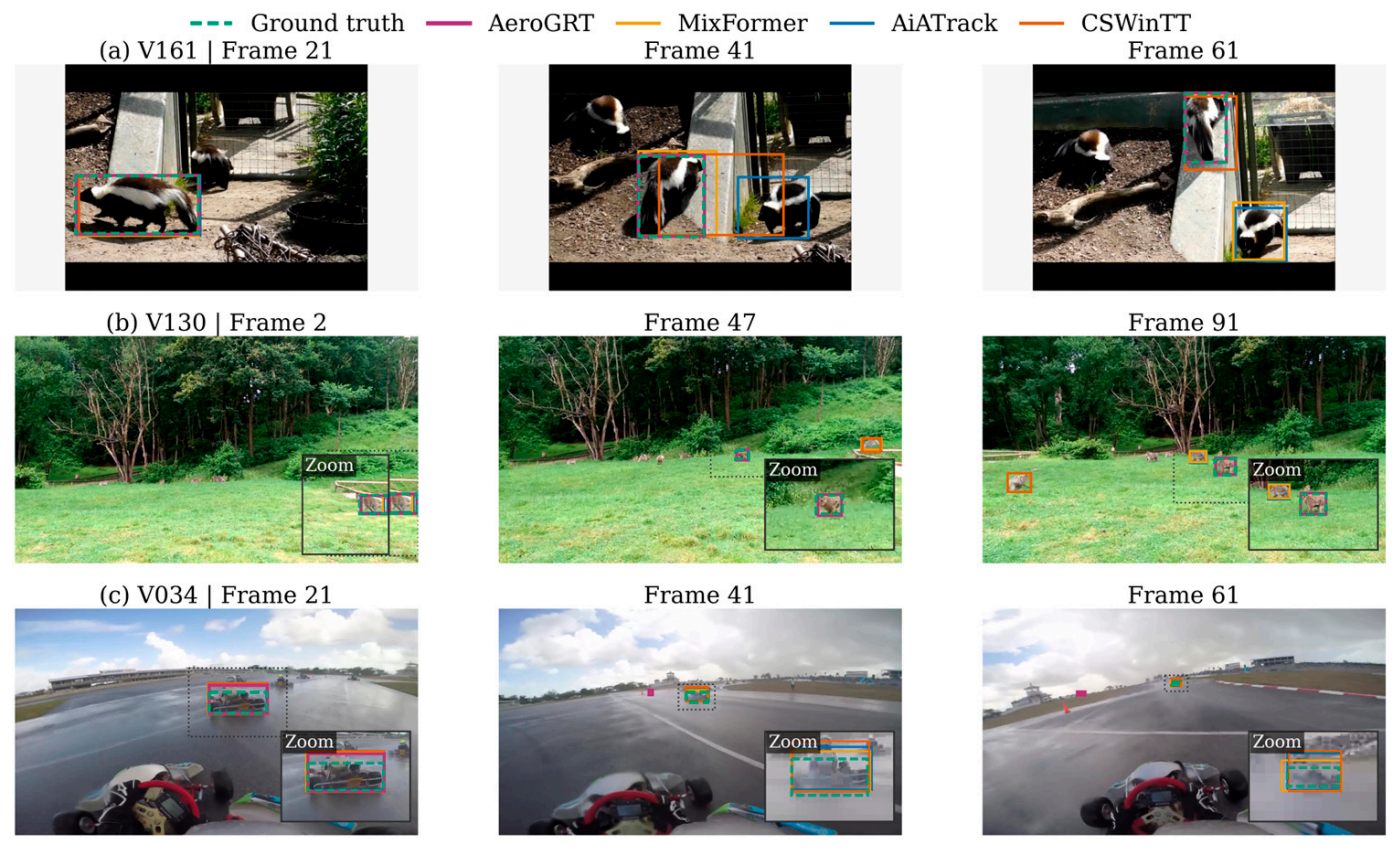
Qualitative tracking results. Green dashed lines are ground truth, purple solid lines are AeroGRT, and other colors denote baselines. The three rows show similar-object interference, small-target tracking, and a failure case.

**Figure 15:**
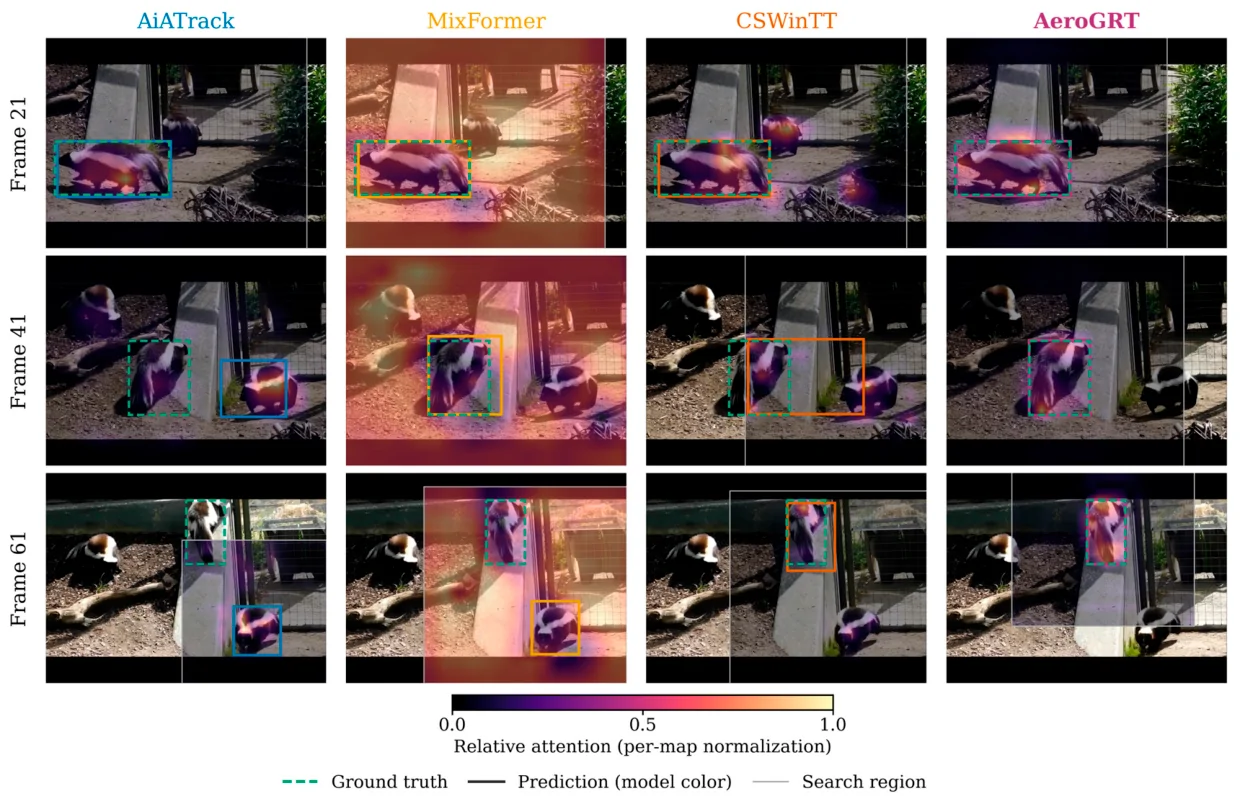
Corresponding attention maps. Brighter regions indicate higher relative attention within each map. Gray borders denote the search region.

To further comprehensively verify the performance of the AeroGRT model, we conduct visualization experiments including quantitative curves, tracking outputs and corresponding attention maps. We compare four pre-trained trackers on a fixed subset of 50 randomly sampled sequences from the AG validation set. All trackers are initialized with the ground-truth bounding box in the first frame, without subsequent ground-truth re-initialization. Metrics are computed over the identical set of 5655 valid non-initialization frames and equally averaged across sequences. The results are illustrated in Figure 13, Figure 14 and Figure 15.

As shown in Figure 13, AeroGRT achieves the best overall localization performance, with an AO of 0.8836, a P20 of 87.56%, and an NP20 of 95.45%. Compared with the strongest baseline, MixFormer, AeroGRT improves AO by 0.0783 and P20 and NP20 by 12.25 and 7.08 percentage points, respectively. The 95% confidence interval for the AO improvement, estimated through paired sequence-level bootstrap resampling, is [0.043, 0.119], supporting a consistent advantage on the evaluated subset.

Figure 14 presents frame-level qualitative results consistent with the quantitative findings. Sequence V161 contains visually similar distractors that challenge target identity preservation. At frame 41, AeroGRT achieves an IoU of 0.970, compared with 0.774, 0.301, and 0 for MixFormer, CSWinTT, and AiATrack, respectively. By frame 61, AeroGRT maintains an IoU of 0.965, whereas AiATrack and MixFormer have drifted away from the annotated target. In V130, AeroGRT successfully localizes the small target at frame 91 with an IoU of 0.922, while all three baselines exhibit zero overlap with the ground truth. These examples illustrate more reliable target retention under similar-instance interference and small-target conditions. Figure 14c also reveals a failure case: as the go-kart recedes in V034, AeroGRT loses the target by frame 41, while MixFormer retains an IoU of 0.714. This case highlights that the aggregate performance advantage does not guarantee reliable tracking across all challenging sequences.

The attention maps in Figure 15 provide a qualitative interpretation of the tracking behavior in V161. At frames 41 and 61, AeroGRT’s high-response regions remain close to the ground-truth target, spatially consistent with its accurate predictions. In contrast, some baseline maps highlight distractor instances or broader background regions. This spatial agreement between attention responses and localization results is consistent with AeroGRT’s stronger target retention in the illustrated sequence.

## 5. Conclusions

This paper presents AeroGRT, a geometry-aware global–local routing Transformer with query-reliability graphs for UAV visual perception. The formulation combines complementary global–local feature extraction, scalar stage weighting of a shared global representation, and foreground–background feature fusion. Cross-axis attention models directional contextual information, while a feature-space consistency mask weights query–key associations. A pyramid-level routing head uses pooled image features and optional altitude metadata to modulate detection confidence without scaling box coordinates. These components are designed to address local-detail preservation, boundary-sensitive representation, feature association, and scale variation in complex UAV scenes.

AeroGRT was evaluated for object tracking on GOT-10k and TrackingNet and for small-object detection on VisDrone and TinyPerson. The GOT-10k ablation results show that IFENM, CAM, and RMA each improve AO over the baseline, and their full combination achieves the highest AO among the tested variants. Visualization experiments on 50 AG validation sequences further supplement the above analyses via performance curves, frame-wise tracking comparisons and attention maps. The examples demonstrate more robust target retention under similar-object interference and small-target scenarios, with attention responses well aligned to annotated targets. Future work will focus on broader multi-dataset evaluation, computational-efficiency optimization, and real-time deployment on UAV edge-computing platforms.

## Figures and Tables

**Table 1:** Representative algorithm families and their relevance to AeroGRT.

Family	Representative Idea	Limitation in UAV Scenes	AeroGRT Response
One-stage detection	Direct dense prediction with real-time inference	Small objects and dense scenes may be underrepresented	Scale–altitude routing and P2–P5 prediction
DETR-style detection	Global set prediction and long-range interaction	Slow convergence and weak local detail in tiny targets	Dual encoder plus local cross-axis attention
Transformer tracking	Template–search interaction and spatio-temporal attention	False matching under background similarity	Query-reliability graph attention
Road distress monitoring	Anisotropic and low-contrast target perception	Strong geometry but task-specific design	Geometry-adaptive feature routing generalized to UAV objects
Edge UAV recognition	On-board inference and low latency	Accuracy–efficiency trade-off under resource limits	Pyramid-level confidence routing with optional altitude metadata

**Table 2:** Relevant datasets used for UAV recognition, tracking, and road monitoring evaluation.

Dataset	Scale and Split	Main Challenge
Got-10k	10,000 training sequences; 180 test sequences	Class diversity and strict one-shot protocol
TrackingNet	30,132 training sequences; 511 test sequences; about 14M boxes	Real-world YouTube videos with 15 attributes
UAV123	123 low-altitude UAV video sequences	Camera motion, altitude variation, scale change
VisDrone2019	6471 train; 548 val; 1610 test-dev; 10 object classes	Dense small objects in 14 cities
TinyPerson	1610 images, commonly split 8:1:1	Tiny pedestrians under high-altitude views
RDD2022-China	4878 China subset images	Ground and drone viewpoints; multiple damage types
UAV-PDD2023	2440 three-channel JPG images	Low contrast and small road damages under UAV imaging
GRDDC2020	21,041 images from Czech Republic, India, and Japan	Cross-country background diversity

**Table 3:** GOT-10k ablation results (unit: %).

Sequence	IFENM	CAM	RMA	AO	SR0.5	SR0.75
1	-	-	-	63.4	74.9	51.4
2	√	-	-	72.9	81.1	71.3
3	-	√	-	72.3	80.2	70.8
4	-	-	√	69.5	76.7	66.4
5	√	√	-	73.4	81.9	72.1
6	-	√	√	72.8	80.9	71.1
7	√	-	√	74.1	82.1	71.9
AeroGRT	√	√	√	75.8	83.9	73.1

**Table 4:** AnisoNet ablation trend used as geometry-aware evidence.

Variant	Core Module	mAP@50 (%)	Efficiency Information	Relevance to AeroGRT
M1	Baseline	86.7	125.64 FPS	Base one-stage detector
M2	LKMSA	89.2	121.48 FPS	Multi-scale large-kernel enhancement
M3	MRDC	90.5	119.37 FPS	Multi-route dynamic semantic modeling
M5	SRBlock	91.8	118.25 FPS	Geometric and serpentine topology modeling
M8	Complete AnisoNet	92.8	115.49 FPS	Synergistic design of attention and geometry

**Table 5:** Statistical significance analysis between the baseline and AeroGRT on GOT-10k.

Method	AO	SR0.5	SR0.75
Baseline	63.5 ± 0.42	74.8 ± 0.36	51.6 ± 0.51
AeroGRT	75.8 ± 0.31	83.9 ± 0.28	73.1 ± 0.35

**Table 6:** Comparative tracking results on GOT-10k (unit: %).

Method	AO	SR0.5	SR0.75	FPS
Baseline	63.4	74.9	51.4	54.5
SiamST	62.1	72.5	51.6	25.81
SimTrack	69.8	78.8	66.0	68.6
CSwinTT	69.4	78.9	65.4	12.4
SLT	67.5	76.5	60.3	50
AiATrack	69.6	80.0	63.2	38
MixFormer-22k	70.7	80.0	67.8	25
SeqTrack	74.8	81.9	72.2	5
AeroGRT	75.8	83.9	73.1	60.9

**Table 7:** Comparison of tracking performance on TrackingNet (unit: %).

Method	AUC	Pnorm	*p*
Baseline	58.1	67.3	54.3
SiamST	65.2	70.9	62.1
SimTrack	83.4	87.4	-
CSwinTT	81.9	86.7	79.5
SLT	82.8	87.5	81.4
AiATrack	82.7	87.8	80.4
MixFormer-22k	83.1	88.1	81.6
SeqTrack	85.5	89.8	85.8
AeroGRT	87.4	91.3	87.6

**Table 8:** Small-object detection gains on VisDrone and TinyPerson.

Method	Dataset	Reference Comparison	mAP50 Gain	mAP50-95 Gain
AeroGRT	VisDrone	YOLOv8n + P2 + P3	+2.8	+1.4
AeroGRT	TinyPerson	YOLOv8n + P2 + P3 + P4	+1.1	+0.2
AeroGRT	VisDrone	YOLOv10n + P2 + P3 + P4 + P5	+3.4	+1.4

## Data Availability

The data presented in this study are not publicly available due to privacy and institutional restrictions. The data may be available from the corresponding author upon reasonable request and with permission of the data provider.
